# Impaired CD27^+^IgD^+^ B Cells With Altered Gene Signature in Rheumatoid Arthritis

**DOI:** 10.3389/fimmu.2018.00626

**Published:** 2018-03-23

**Authors:** Fanlei Hu, Wei Zhang, Lianjie Shi, Xu Liu, Yuan Jia, Liling Xu, Huaqun Zhu, Yingni Li, Dakang Xu, Liwei Lu, Xiaoyan Qiu, Wanli Liu, Junjie Qiao, Yongfu Wang, Zhanguo Li

**Affiliations:** ^1^Department of Rheumatology and Immunology, Peking University People’s Hospital, Beijing Key Laboratory for Rheumatism Mechanism and Immune Diagnosis (BZ0135), Beijing, China; ^2^Department of Rheumatology and Immunology, First Hospital Affiliated to Baotou Medical College, Inner Mongolia Key Laboratory of Autoimmunity, Baotou, China; ^3^Department of Rheumatology and Immunology, Peking University International Hospital, Beijing, China; ^4^Department of Molecular and Translational Science, Faculty of Medicine, Hudson Institute of Medical Research, Monash University, Melbourne, VIC, Australia; ^5^Department of Pathology, The University of Hong Kong, Hong Kong, Hong Kong; ^6^Department of Immunology, School of Basic Medical Science, Peking University, Beijing, China; ^7^MOE Key Laboratory of Protein Sciences, Collaborative Innovation Center for Diagnosis and Treatment of Infectious Diseases, School of Life Sciences, Institute for Immunology, Tsinghua University, Beijing, China; ^8^Department of Orthopedics, Xuanwu Hospital, Capital Medical University, Beijing, China; ^9^Peking-Tsinghua Center for Life Sciences, Beijing, China; ^10^State Key Laboratory of Natural and Biomimetic Drugs, School of Pharmaceutical Sciences, Peking University, Beijing, China

**Keywords:** rheumatoid arthritis, innate-like B cells, CD27^+^IgD^+^ B cells, natural IgM, gene signature

## Abstract

Natural antibodies, particularly natural IgM, are proved to play indispensable roles in the immune defenses against common infections. More recently, the protective roles of these natural IgM were also recognized in autoimmune diseases. They are mainly produced by B-1 and innate-like B cells (ILBs). Human CD19^+^CD27^+^IgD^+^ B cells, also termed as un-switched memory B cells, were proposed to be a kind of ILBs. However, functional features and characteristics of these cells in rheumatoid arthritis (RA) remained poorly understood. In this study, we found that human CD27^+^IgD^+^ B cells could produce natural antibody-like IgM. Under RA circumstance, the frequencies of these cells were significantly decreased. Moreover, the IgM-producing capacities of these cells were also dampened. Interestingly, the BCR repertoire of these cells was altered in RA, demonstrating decreased diversity with preferential usage alteration from VH3-23D to VH1-8. Single cell sequencing further revealed the proinflammatory biased features of these cells in RA. These CD27^+^IgD^+^ B cells were negatively correlated with RA patient disease activities and clinical manifestations. After effective therapy with disease remission in RA, these cells could be recovered. Taken together, these results have revealed that CD27^+^IgD^+^ B cells were impaired in RA with dysfunctional features, which might contribute to the disease perpetuation.

## Introduction

Rheumatoid arthritis (RA) is a chronic systemic inflammatory disease characterized by hyperplasia of synovial fibroblasts and variable degrees of bone and cartilage erosion, leading to impairment of joint function (1). The pathogenesis remains to be fully elucidated.

A key characteristic of RA is the occurrence of antibodies against post-translationally modified self-antigens. Citrullination and anti-citrullinated protein antibodies (ACPAs) have been studied extensively (2). These autoantibodies could be detected as long as 14 years before the onset of the disease (3). Moreover, the high-affinity IgG autoantibodies contribute directly to RA pathogenicity by multiple mechanisms. For example, ACPAs were proved to induce osteoclastogenesis and bone-resorption directly *via* an IL-8-dependent mechanism, thus exacerbating the disease progression (4). Whereas, some IgG autoantibodies are pathogenic, mounting evidence indicates that specialized classes of IgM natural antibodies exist, with properties that can oppose the development of RA.

Natural IgM, in contrast to immune IgM, is produced in the absence of pathogen encounters (5). It constitutes the majority of total circulating IgM. Most of natural IgM are germline-encoded and polyreactive, binding with low affinity to a number of different antigens. Besides providing host defense against bacterial, viral, and fungal microbial infections, these natural IgM could help to clear the apoptotic cells as well as the neo-antigens, and suppress the innate inflammation, thus sustaining the immune homeostasis and protecting the body from autoimmunity (6).

B-1 cells and innate-like B cells (ILBs) are proved to be the major producer of natural IgM (7). The source and features of B-1 and ILBs have been well studied in mice, whereas they remained controversial in human. CD19^+^CD27^+^IgD^+^ B cells which are also termed as un-switched memory B cells were proposed to be a kind of human ILBs (8). The pathogenic roles of B cells, particularly plasmablasts and switched memory B cells, have been studied well, which contribute to the development of RA by producing ACPAs autoantibodies, presenting auto-antigen and secreting cytokines (9). The impairment of regulatory B cells (Breg), including IL-10-producing Breg (B10) and granzyme B-producing Breg further improved our understanding of the role of B cells in RA pathogenesis (10, 11). Nevertheless, the characteristics of CD27^+^IgD^+^ B cells and their potential role in RA are largely unknown.

In this study, we systemically characterized CD27^+^IgD^+^ B cells in RA, revealing their numerical deficiency, natural antibody-like IgM-producing capacity impairment, BCR repertoire alteration as well as gene expression profile bias that would exacerbate the disease progression.

## Materials and Methods

### Patients and Tissue Specimens

76 RA patients [27 receiving anti-TNF-α monoclonal antibody therapy (12 etanercept, 11 infliximab, 4 adalimumab), including 7 pairs before and after therapy, Table 1], 10 OA patients (8 female and 2 male), as well as 78 age- and sex-matched healthy volunteers were enrolled in the study. All patients fulfilled the American College of Rheumatology 2010 criteria for RA, and 1995 criteria for OA. The study was approved by Institutional Medical Ethics Review Board of Peking University People’s Hospital, and all the participants provided written informed consent.

**Table 1:** Clinical characteristics of RA patients.

Characteristics	RA (*n* = 76)
Age, mean (range), years	57.7 (33–84)
Sex, no. female/male	61/15
Duration, mean (range), month	120.09 (1–372)
Tender joint count, mean (range) of 28 joints	17 (2–28)
Swollen joint count, mean (range) of 28 joints	15.8 (0–28)
DAS28, mean (range)	7.47 (4.81–9.22)
ESR, mean (range), mm/h	56.29 (10–108)
CRP, mean (range), mg/l	38.54 (1.97–132)
RF, no. positive/no. negative/no. nd	55/18/3
ACPA, no. positive/no. negative/no. nd	62/12/2

*ESR, erythrocyte sedimentation rate; CRP, C-reactive protein; RF, rheumatoid factor; ACPA, anti-cyclic citrullinated peptide antibody; RA, rheumatoid arthritis*.

### Flow Cytometry Analysis and Sorting

For CD27^+^IgD^+^ B cell detection, 100 µl fresh whole blood cells were stained with APC-CY7-conjugated anti-CD19 (Biolegend, San Diego, CA, USA), APC-conjugated anti-CD27 (eBioscience, San Diego, CA, USA), and FITC-conjugated anti-IgD (eBioscience). Then the red blood cells were lysed using the RBC lysis buffer (MultiSciences, Hangzhou, China) and the left cells were analyzed on FACS Aria II. Dead cell exclusion was performed by scatter profiles and 7-AAD staining during all the flow cytometric analyses.

For CD27^+^IgD^+^ B cell sorting, peripheral blood mononuclear cells (PBMCs) were isolated from fresh blood samples using Ficoll density-gradient centrifugation, and then were stained as described above. After that, the aimed cells were harvested into the collection solution (RPMI 1640 supplemented with 10% FBS and 2% antibiotics) using FACS Aria II flow cytometry sorter according to the manufacturer’s instructions. The purified cells were further analyzed after sorting, the purity of which was 95–99%.

### RT-PCR and Realtime PCR

Total RNA was extracted from cells using RNeasy Mini Kit (Qiagen, Hilden, Germany), and then was reverse transcribed into cDNA with the RevertAid First Strand cDNA synthesis kit (Fermentas, Glen Burnie, MD, USA) according to the manufacturer’s instructions. The resulting cDNA was subjected to PCR and realtime PCR analyses (primers as in Table S1 in Supplementary Material). For realtime PCR, gene expression was quantified relative to the expression of the housekeeping gene GAPDH as well as β-actin, and was normalized to control by standard 2^−ΔΔCT^ calculation as previously described (12).

### ELISPOT Analysis

ELISPOT was performed using the ELISpot^PLUS^ Human IgM Kit (MABTECH AB, Sweden) as described previously (13). Briefly, 1 × 10^4^ flow cytometry-sorted CD27^+^IgD^+^ B cells as well as other B cell subsets from healthy individuals or RA patients were subjected to IgM detection in the presence of anti-CD40 (3 µg/ml, eBioscience) and CpG (10 µg/ml, Invivogen, San Diego, CA, USA) for 24 h. The results were analyzed on an ImmunoSpot Analyzer (Cellular Technology Ltd., Shaker Heights, OH, USA).

### BCR Repertoire Sequencing

To amplify the variable region of IgM heavy chain, semi-nested PCR was performed using the universal primers as in Table S1 in Supplementary Material, with bulk sorted CD27^+^IgD^+^ B cells as the template. Generally, for the first round amplification, VH3, VH4, VH5, VH6 (up-stream), and CμCH1 (down-stream) were used, while for the second round amplification, VH3, VH4, VH5, VH6 (up-stream), and J2 (down-stream) were used. The PCR products were separated on 1% agarose gel by electrophoresis and were visualized by GoldView II staining (Solarbio Biocompany, Beijing, China). The aimed band was extracted from the gels and further purified using the kits (DP209) from TIANGEN BIOTECH Co., Ltd., (Beijing, China). Then the final products were used for single clone sequencing as described previously (14). Briefly, the PCR products were cloned into T-EASY vector, transformed into TOP10 bacteria, and planted into the amplicilin-containing LB plates. Then ~50 random single clones were harvested, verified, then were subjected to the DNA sequencing. The VDJ rearrangements were further analyzed using the Ig blast in NCBI website.

### Single Cell Sequencing

CD27^+^IgD^+^ B cells were sorted from three active RA patients (DAS28 > 5.1) and three healthy controls by flow cytometry (~1,000 cells per sample), then were subjected to single cell sequencing accomplished by Beijing Novogene Bioinformatics Technology Co., Ltd. (Beijing, China). The raw data were processed to clean expression signals. The significantly differentially expressed (DE) genes (*P* < 0.05, at least twofold changes) were first analyzed for hierarchical clustering. To interpret their biological meanings, these DE transcripts were further functionally categorized according to the gene ontology (GO) and Kyoto Encyclopedia of Genes and Genomes (KEGG) databases. *P* values were calculated based on hypergeometric distribution analysis.

### Polyreactivity ELISA

To analyze the reactivity of CD27^+^IgD^+^ B cells-derived IgM, microtiter plates were coated with the well-studied RA autoantigens, including ssDNA, fibrinogen, vimentin, and collagen II (Sigma, 10 µg/ml each). BSA was chosen as the control. 100 µl culture supernatants of the CD27^+^IgD^+^ B cells (cultured as described above) from both healthy individuals and RA patients were tested, with the medium only as the background control. Biotin-conjugated goat anti-human IgM and HRP-labeled streptavidin (BETHYL, Montgomery, TX, USA) were used for the IgM detection, with TMB (Neobioscience Technology, Beijing, China) as the substrate. OD450 was measured using a microplate reader (Bio-Tek, Winooski, VT, USA). The reactivity was demonstrated as (OD450_cell culture supernatants_ − OD450_medium control_).

### Statistics

All statistical calculations were performed using the statistical software program SPSS 17.0 (SPSS, Chicago, IL, USA). Differences between various groups were evaluated by the Student’s *t*-test, paired *t*-test, one-way ANOVA test, or spearman test, and were statistically significant at *P* < 0.05.

## Results

### Human CD27^+^IgD^+^ B Cells Could Produce Natural Antibody-Like IgM

**Figure 1:**
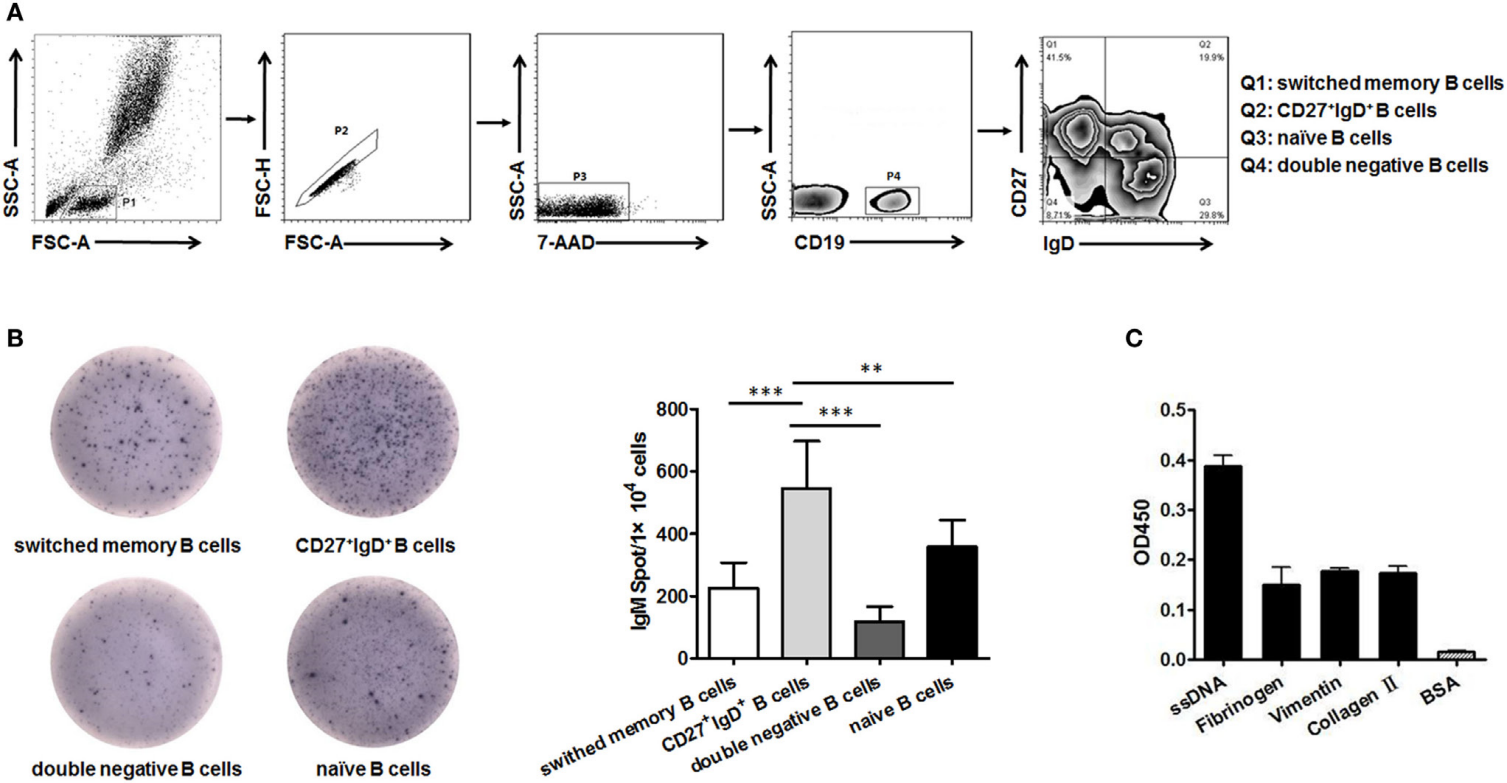
Human CD27^+^IgD^+^ B cells could produce natural antibody-like IgM. **(A)** Gating strategy for identifying CD27^+^IgD^+^ B cells (Q2) as well as double negative B cells (Q4, CD27^−^IgD^−^), naïve B cells (Q3, CD27^−^IgD^+^), and switched memory B cells (Q1, CD27^+^IgD^−^) according to CD27 and IgD expression. **(B)** CD27^+^IgD^+^ B cells as well as the other three B cell subsets were sorted from healthy volunteer peripheral blood (*n* = 5) by flow cytometry, and then were subjected to IgM ELISPOT analysis (one-way ANOVA, **P < 0.01, ***P < 0.001). **(C)** Flow cytometry-sorted healthy volunteer CD27^+^IgD^+^ B cells (*n* = 5) were cultured *in vitro* for 3 days in the presence of anti-CD40 (3 µg/ml) and CpG (10 µg/ml), then the cell culture supernatants were collected for polyreactivity ELISA against different rheumatoid arthritis autoantigens, including ssDNA, fibrinogen, vimentin, and collagen II. BSA was chosen as the control. The results were presented as mean ± SEM.

B cells could be divided into four subsets based on their expression of CD27 and IgD, naïve B cells (CD27^−^IgD^+^), un-switched memory B cells (CD27^+^IgD^+^), switched memory B cells (CD27^+^IgD^−^), and double negative B cells (CD27^−^IgD^−^) as demonstrated in (Figure 1A). We next compared their capacity in producing IgM by ELISPOT. The result showed that CD27^+^IgD^+^ B cells were the most competent cells in producing IgM without antigen stimulation, with naïve B cells as the second competent ones (Figure 1B). To explore the natural antibody properties of CD27^+^IgD^+^ B cells derived IgM, polyreactivity ELISA, as well as the BCR repertoire analysis of the VH segments were performed. It was shown that these CD27^+^IgD^+^ B cells-derived IgM could bind with different RA autoantigens with low affinity, including ssDNA, fibrinogen, vimentin, and collagen II (Figure 1C). More important, the variable region of these IgM heavy (μ) chain (VH) showed preferential usage as shown below, yet with low level of somatic hypermutation (3.18 ± 1.58%). Taken together, these results showed that human CD27^+^IgD^+^ B cells could produce natural antibody-like IgM without antigen encounter, demonstrating polyreactivity with low affinity and VH preferential usage with low mutation.

### CD27^+^IgD^+^ B Cells Were Significantly Decreased in RA, Correlating With the Disease Manifestations

**Figure 2:**
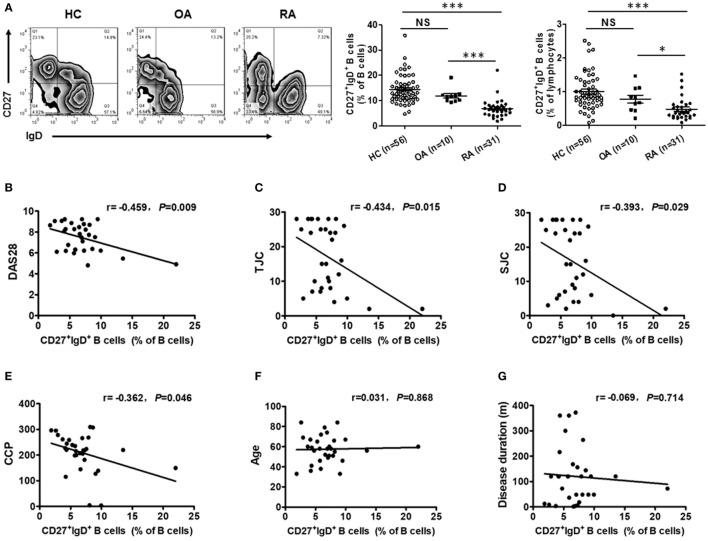
Decreased frequencies of CD27^+^IgD^+^ B cells in rheumatoid arthritis (RA). **(A)** Flow cytometric analysis of the frequencies of CD27^+^IgD^+^ B cells in the peripheral blood of 56 healthy donors, 31 RA patients as well as 10 non-autoimmune osteoarthritis patients. The representative flow charts and the statistical results (including CD27^+^IgD^+^ B cells% in B cells and CD27^+^IgD^+^ B cells% in lymphocytes) were shown (one-way ANOVA, *P < 0.05, ***P < 0.001, NS, not significant). And also, the correlation of CD27^+^IgD^+^ B cells with RA patient clinical manifestations, including the disease activity scores [DAS28, **(B)**], the tender joint counts [TJC, **(C)**], the swollen joint counts [SJC, **(D)**], the anti-cyclic citrullinated protein antibodies [anti-CCP, **(E)**], the age **(F)**, and the disease duration **(G)** was analyzed by the Spearman test (*P < 0.05).

To reveal the role of CD27^+^IgD^+^ B cells in RA pathogenesis, we first compared their frequencies with RA patients and healthy individuals. As shown in Figure 2A, compared with the 56 healthy volunteers, the frequencies of CD27^+^IgD^+^ B cells were significantly decreased in the peripheral blood of 31 RA patients. However, their frequencies were not declined in the OA patients, a degenerative, but not in inflammatory arthritis. Moreover, the frequencies of these CD27^+^IgD^+^ B cells were inversely correlated with RA patient disease activities (DAS28) and the clinical manifestations, including the tender joint counts (TJC), swollen joint counts (SJC), and the anti-cyclic citrullinated protein (anti-CCP) antibodies, but not the ages and disease duration (Figures 2B–G; Table 2). These data demonstrated that CD27^+^IgD^+^ B cells were numerically deficient under RA circumstance.

**Table 2:** Correlation analysis of CD27^+^IgD^+^ B cells with rheumatoid arthritis patient clinical manifestations.

Clinical manifestation	*r*	*P*
Age	0.031	0.868
Duration	−0.069	0.714
DAS28	**−0.459**	0.009**
Tender joint counts	**−0.434**	0.015*
Swollen joint counts	**−0.393**	0.029*
CCP	**−0.362**	0.046*
Rheumatoid factor	−0.154	0.407
ESR	−0.019	0.918
C-reactive protein	−0.295	0.108
IgA	−0.099	0.596
IgG	−0.159	0.392
IgM	−0.188	0.311

*Bold font indicates having statistical significance*.

*P < 0.05, **P < 0.01.

### CD27^+^IgD^+^ B Cells Demonstrated Dampened IgM-Producing Competency in RA

**Figure 3:**
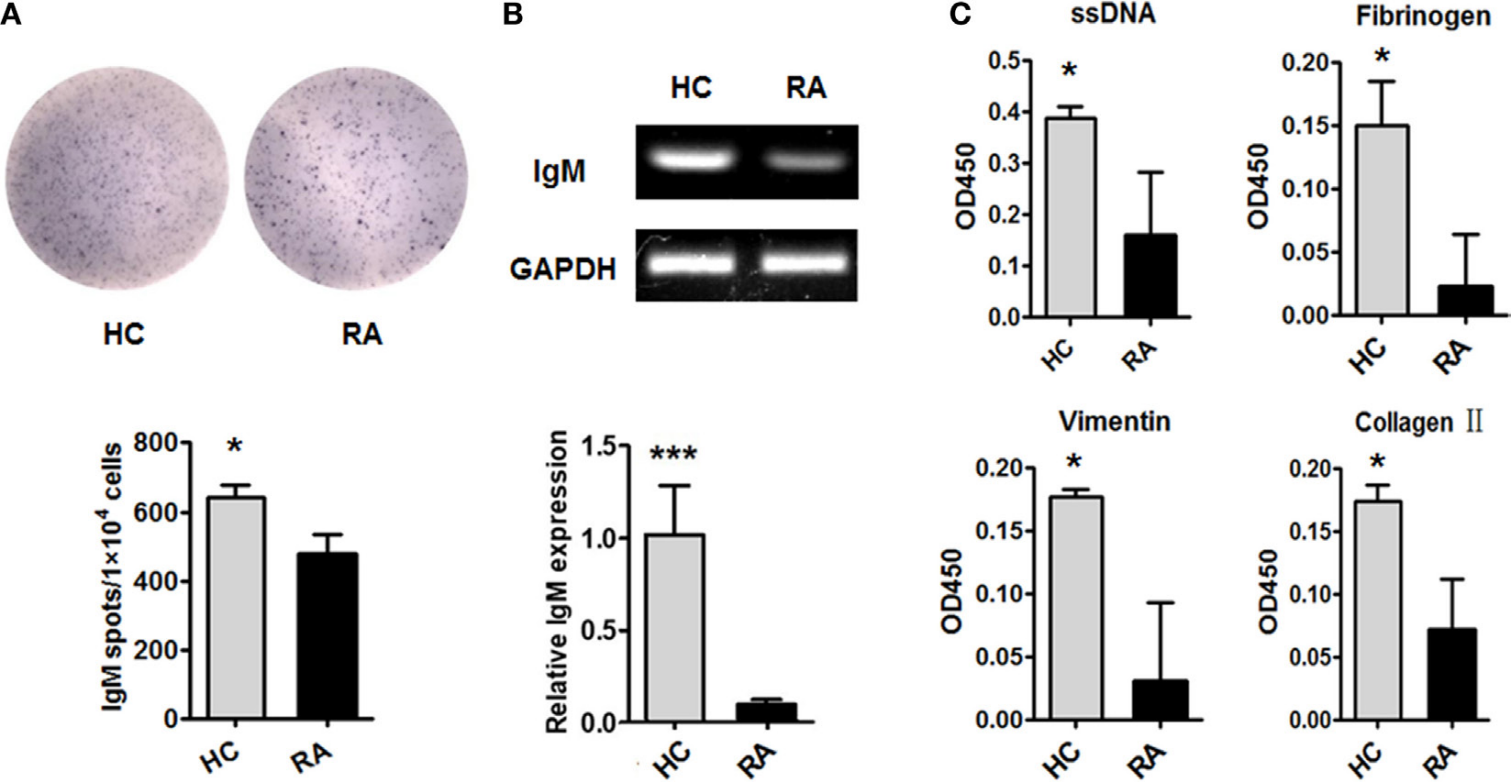
Dampened IgM-producing capacities of CD27^+^IgD^+^ B cells in rheumatoid arthritis (RA). Flow cytometry-sorted CD27^+^IgD^+^ B cells from six healthy donors and five active RA patients (DAS28 > 5.1) were subjected to ELISPOT **(A)**, PCR and realtime PCR **(B)**, analyses of IgM. The representative charts as well as the statistical results were shown (*t*-test, *P < 0.05, ***P < 0.001). **(C)** And also, the culture supernatants of equal number of CD27^+^IgD^+^ B cells from healthy donors (*n* = 6) and active RA patients (DAS28 > 5.1, *n* = 5) were collected for polyreactivity ELISA against RA autoantigens, including ssDNA, fibrinogen, vimentin, and collagen II (*t*-test, *P < 0.05). The results were presented as mean ± SEM.

We then examined the production of IgM by CD27^+^IgD^+^ B cells in RA patients. CD27^+^IgD^+^ B cells from active RA patients (DAS28 > 5.1) and healthy individuals were isolated by flow cytometry sorting and subjected to ELISPOT, RT-PCR, and QPCR analyses. ELISPOT analysis showed that under RA circumstance, the IgM-producing capacities of these cells were dampened (Figure 3A). RT-PCR and QPCR analyses further confirmed that the IgM transcripts of these CD27^+^IgD^+^ B cells were significantly decreased in RA (Figure 3B). To further characterize their IgM-producing competency, we compared the binding capacities of these IgM with different RA autoantigens. As shown in Figure 3C compared with healthy controls, RA patient CD27^+^IgD^+^ B cells-derived IgM showed fundamentally declined binding with ssDNA, fibrinogen, vimentin, and collagen II, the well-known RA autoantigens. Taken together, these results revealed that CD27^+^IgD^+^ B cells were functionally impaired in producing IgM in RA.

### The BCR Repertoire of CD27^+^IgD^+^ B Cells Showed Decreased Diversity With Different Preferential Usage in RA

**Figure 4:**
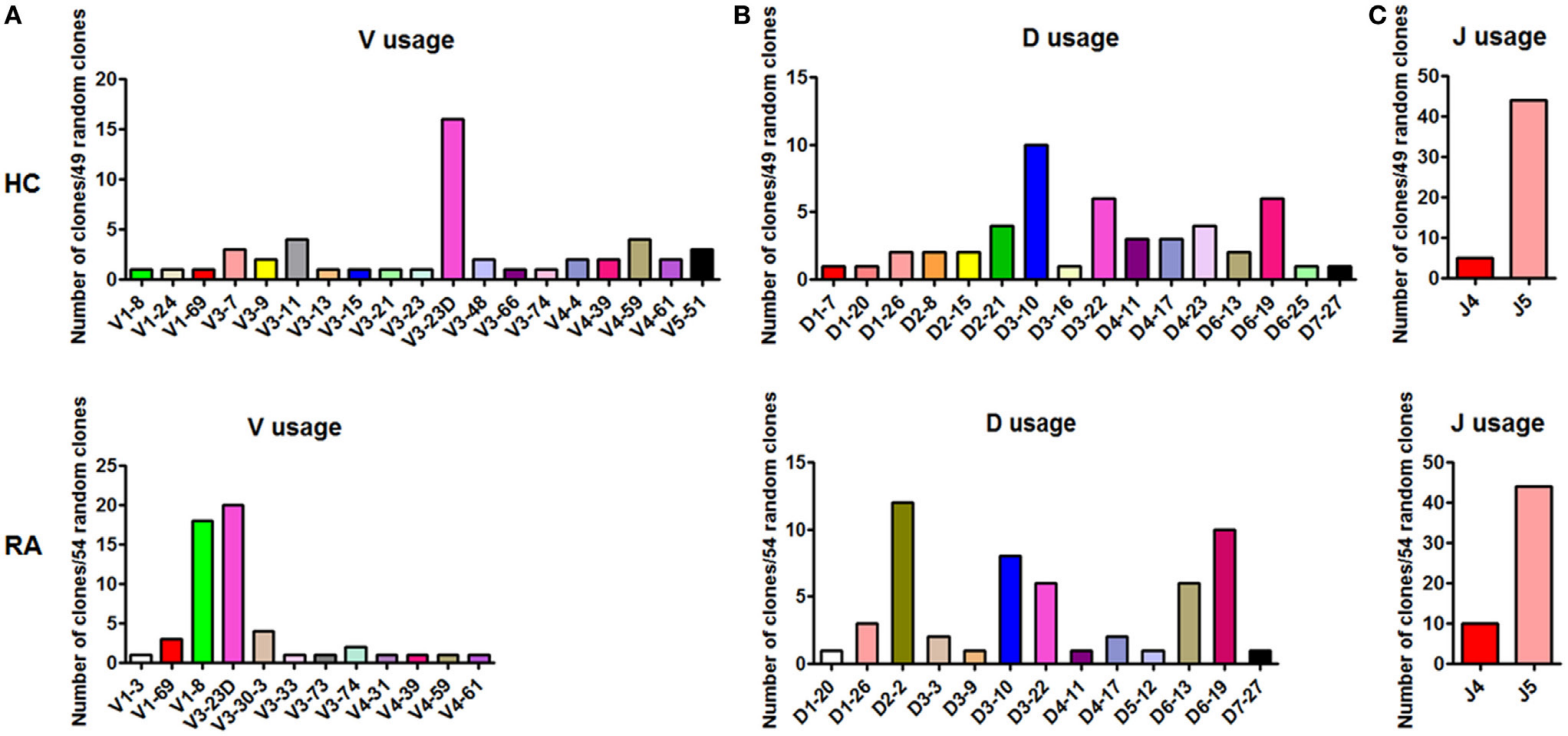
BCR repertoire analysis of CD27^+^IgD^+^ B cells both in healthy individuals and rheumatoid arthritis (RA) patients. CD27^+^IgD^+^ B cells from both healthy individuals (*n* = 11) and active RA patients (DAS28 > 5.1, *n* = 10) were sorted by flow cytometry and subjected to the PCR amplification of the variable region of the Igμ chain using the universal primers. Then the PCR products were purified and cloned into the T-EASY vector for the following single clone sequencing (~50 random clones). The VDJ rearrangements were further analyzed using the Ig blast database in the NCBI website. **(A)**: V usage; **(B)**: D usage; **(C)**: J usage.

Followingly, we analyzed the BCR repertoire of CD27^+^IgD^+^ B cells both in healthy individuals and RA patients by single clone sequencing. Compared with healthy controls, RA patient CD27^+^IgD^+^ B cells-derived IgM also showed preferential usage in the variable region of the heavy (μ) chain (VH). However, unlike the healthy individuals with VH3-23D as the preferential usage, RA patients demonstrated two preferential usages, VH3-23D and VH1-8 (Figure 4A). Yet, little difference was shown for the D and J segments (Figures 4B,C). Notably, the VH usage showed decreased diversity in RA patients (Figures 4A,B), which might contribute to the declined antigen binding.

### CD27^+^IgD^+^ B Cells Revealed Expression Profile of Proinflammatory Bias in RA

**Figure 5:**
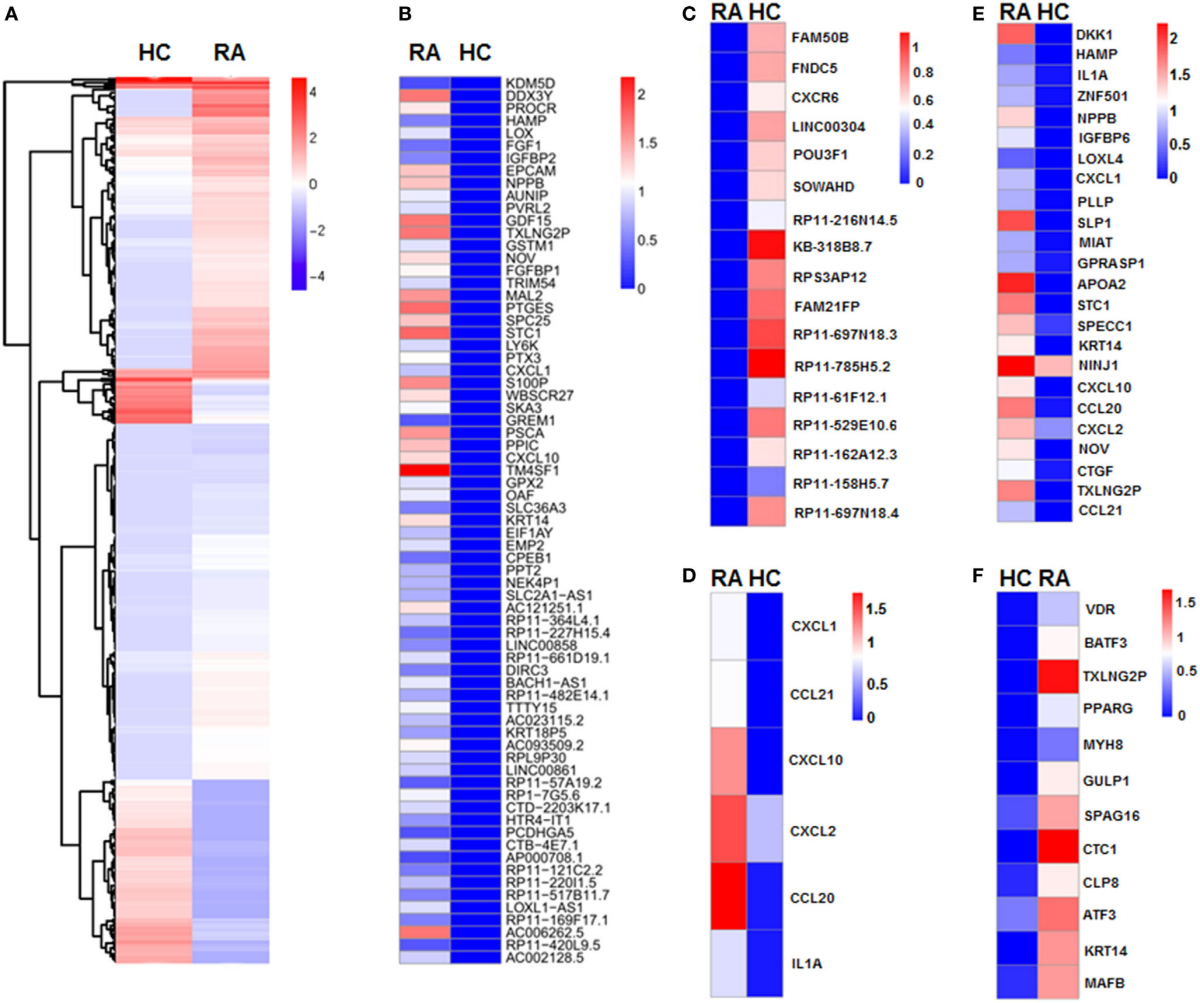
Single cell sequencing of CD27^+^IgD^+^ B cells in healthy controls and rheumatoid arthritis (RA) patients. **(A)** Unsupervised clustering analysis of differentially expressed genes of CD27^+^IgD^+^ B cells in RA and healthy individuals based on single cell sequencing data. Blue and red represent low and high expression levels, respectively. **(B)** Heat-map of clustering analysis of 71 genes uniquely expressed in RA, but not in healthy control CD27^+^IgD^+^ B cells. **(C)** Heat-map of clustering analysis of 17 genes uniquely expressed in healthy control, but not in RA CD27^+^IgD^+^ B cells. **(D)** Gene ontology (GO) analysis of differentially expressed genes with regards to chemokine activity and chemokine receptor binding, cytokine activity and cytokine receptor binding as well as immune response. **(E,F)** GO analysis of differentially expressed genes with regards to extracellular region **(E)** and transcriptional factors **(F)**, respectively.

We next revealed the specific transcriptome and gene signature of CD27^+^IgD^+^ B cells in RA and in healthy individuals by single cell sequencing. We identified a total of 401 DE transcripts, with 295 upregulated and 106 downregulated in RA (*P* < 0.05, at least twofold changes). These genes clearly distinguish active RA patients with healthy controls which were visualized in a hierarchical clustering diagram (Figure 5A). In particular, 71 genes were uniquely expressed in RA (Figure 5B), while 17 genes were uniquely expressed in HC (Figure 5C).

To elucidate the potential functional difference of CD27^+^IgD^+^ B cells in RA and HC, we further categorized the DE genes according to the GO analysis, which includes three GO systems: molecular function, biological process, and cellular component. Results showed that the DE genes in CD27^+^IgD^+^ B cells were enriched in several common ontologies, including chemokine activity and chemokine receptor binding, cytokine activity and cytokine receptor binding, immune response (Figure 5D), extracellular region (Figure 5E), transcriptional factors (Figure 5F), receptor binding, and cell growth. Especially, RA patient CD27^+^IgD^+^ B cells expressed higher levels of chemokines and cytokines, including chemokine (C-C motif) ligand (CCL) 21, CCL20, chemokine (C-X-C motif) ligand (CXCL) 10, CXCL1, CXCL2, and interleukin 1α, demonstrating features of proinflammatory bias (Figure 5D). KEGG pathway analysis further revealed the enrichment of DE genes in the chemokine signaling pathway, cytokine–cytokine receptor interaction pathway, TNF signaling pathway, and rheumatoid arthritis pathway, confirming the pathogenic features of CD27^+^IgD^+^ B cells in RA.

### Recovery of CD27^+^IgD^+^ B Cells in RA Patients With Disease Remission After Therapy

**Figure 6:**
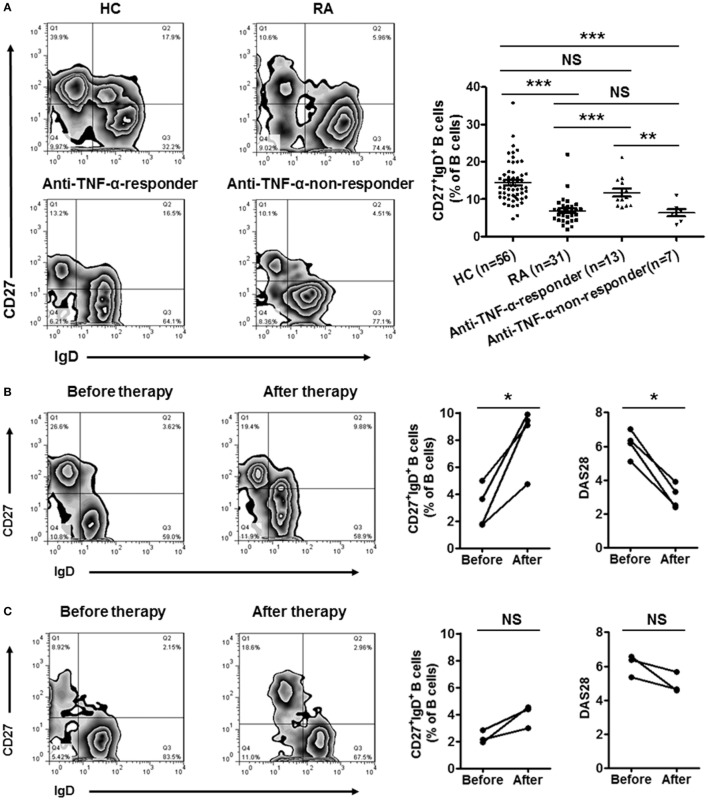
Recovery of CD27^+^IgD^+^ B cells in rheumatoid arthritis (RA) patients with disease remission after therapy. **(A)** The frequencies of CD27^+^IgD^+^ B cells in RA patients receiving anti-TNF-α mAb therapy (*n* = 20, 13 responders and 7 non-responders) or not (*n* = 31) were analyzed by flow cytometry. The representative flow charts and the statistical results were shown (one-way ANOVA, **P < 0.01, ***P < 0.001, NS, not significant). **(B,C)** Seven RA patients (DAS28 > 5.1) before and after anti-TNF-α antibody treatment were recruited in the study. The frequencies of CD27^+^IgD^+^ B cells as well as the disease activity score DAS28 were analyzed (Paired *t*-test, *P < 0.05, NS, not significant). **(B)** 4 good responders, **(C)** 3 poor responders.

Since CD27^+^IgD^+^ B cells were decreased in RA patients and negatively correlated with the disease activity, we further evaluated whether these cells would be recovered after effective therapy. We first detected the frequencies of CD27^+^IgD^+^ B cells in RA patients receiving anti-TNF-α mAb treatment (*n* = 20) or not (*n* = 31). In the 20 RA patients receiving anti-TNF-α biologics therapy, 13 showed good responses while 7 showed poor responses. As shown in Figure 6A, for the RA patients with good responses, the frequencies of CD27^+^IgD^+^ B cells increased significantly, almost to the normal levels. However, the RA patients with poor responses demonstrated little change of CD27^+^IgD^+^ B cells. Yet, another possibility might be that high frequencies of CD27^+^IgD^+^ B cells at baseline could predict treatment success with anti-TNF-α therapy.

To tease out these two possibilities, a follow-up study was carried out, with recruitment of seven RA patients before and after anti-TNF-α therapy. In the 4 good responders out of the 7 patients, the frequencies of CD27^+^IgD^+^ B cells were increased significantly (Figure 6B). However, in the 3 poor responders, these B cells were not rescued (Figure 6C).

All these results suggested that the impaired CD27^+^IgD^+^ B cells in RA patients could be recovered after effective therapy, indicating their deficiency might be correlated with the development of RA.

## Discussion

In this study, we revealed that in RA CD27^+^IgD^+^ B cells were numerically decreased and functionally impaired in producing natural antibody-like IgM that could bind with RA autoantigens, correlating with the patient clinical features. BCR repertoire and gene expression profile analyses further revealed their IgM VH preferential usage alteration and proinflammatory bias in RA. After effective therapy, these CD27^+^IgD^+^ B cells could get recovered.

The protective role of natural IgM has been recognized, since the construction of the secreted (s)IgM-deficient mice (5). These mice were more susceptible to bacterial infections and 70% of the mice died in an acute peritonitis model. Followingly, these natural IgM were proved to play an important non-redundant role in the first line defense against bacterial, viral, and fungal infections (6). Thereafter, natural IgM has been revealed to demonstrate more protective functional properties. They could recognize the apoptotic cells and self-antigens, especially the oxidation-associated neo-antigens, and enhance their phagocytic clearance, therefore, have been proposed to provide important homeostatic “house-keeping” functions (15). Mice deficient in natural IgM displayed increased susceptibility to autoimmunity, spontaneously produced IgG autoantibodies to nuclear antigens, and developed lupus with aging (16, 17). These natural antibodies can also suppress the key signaling pathways in the inflammatory responses mediated by the innate immune system, particularly the mitogen-activated protein kinase (MAPK) pathways (18). Among these natural antibodies, anti-phosphorylcholine (anti-PC) natural IgM were the most extensively studied (19, 20).

Clinical surveys indicated that natural IgM might be involved in the protection against developing cardiovascular complications, and their deficiency correlated with the incidence of systemic autoimmune diseases. SLE patients were reported to demonstrate lower levels of anti-PC natural IgM, reductions of which correlated with duration of the disease (21). Lower levels of anti-PC natural IgM were also reported to be associated with more frequent cardiovascular events in patients with SLE (22). Natural IgM were also proved to be involved in the pathogenesis of RA. More important, administration of the anti-PC natural IgM could ameliorate or even prevent the clinical and histological features of experimental inflammatory arthritis models, including the collagen antibody-induced arthritis (CAIA) and collagen-induced arthritis (CIA) (23). RA patients with lower titers of anti-PC natural IgM were more likely to experience cardiovascular events (24). Yet, till now little is known about natural IgM in human RA.

Innate-like B cells and B-1 cells were proved to be the major producer of natural IgM. Although well characterized in mice, large remains unknown about the source and features of B-1 and ILBs in human. In this study, we proved that CD27^+^IgD^+^ B cells demonstrated the characteristics of ILBs in human. They could spontaneously secrete IgM that were polyreactive and low affinitive, with preferential VH usage. However, their VH demonstrated low level of mutation, distinguishing them from true natural IgM. Therefore, we termed these CD27^+^IgD^+^ B cells-derived IgM as natural antibody-like IgM. Moreover, we found that CD27^+^IgD^+^ B cells were significantly decreased under RA circumstance. One possible mechanism might be that these B cells were exhausted by the RA inflammatory milieu. RA is a complexed disease with series of pro-inflammatory cytokine secretion, including TNF-α, IL-1β, IL-6, IFN-γ, and IL-17. Previous studies showed that pro-inflammatory cytokines, particularly TNF-α disrupted the stability of regulatory T cells (Treg), which could even drive the pathogenic conversion of these cells in autoimmune diseases (25). Our recent studies also revealed that TNF-α could stimulate the pathogenic conversion of regulatory B10 cells into RANKL-producing cells (10). As a new B cell subset with potential regulatory functions, CD27^+^IgD^+^ B cells might also be inhibited by RA pro-inflammatory cytokines, thus inducing their numerical demission. Indeed, our preliminary data showed that CD27^+^IgD^+^ B cells were diminished after *in vitro* co-culture with TNF-α (data not shown), yet the detailed mechanism as well as the other cytokines’ effects need to be further studied.

In this study, we also found that the BCR repertoire of CD27^+^IgD^+^ B cells was altered in RA. Compared with healthy controls, the variable region of the CD27^+^IgD^+^ B cells-derived IgM μ chain showed much narrower spectrum. The decreased TCR or BCR diversity has been observed in cancers and autoimmune diseases, which may contribute to the development of the diseases (26). The decreased diversity of IgM μ chain variable region of CD27^+^IgD^+^ B cells might also contribute to RA disease progression. Moreover, different preferential usages of the IgM variable region were also observed for these cells in RA, revealing differential patterns. These biased preferential usages of VH region might demonstrate different binding ability with autoantigens. Our hypothesis is that, under RA circumstance, the altered VH1-8, compared with VH3-23D in the healthy controls, might show lower affinity with RA autoantigens, thus failing to eliminate them efficiently. The accumulated autoantigens would eventually break the threshold of autoimmunity and lead to the production of autoantibodies. Yet, to further confirm this, monoclonal antibodies carrying the corresponding VH segments should be prepared to compare their binding abilities, which will be performed in our following studies.

Single cell sequencing data showed that RA CD27^+^IgD^+^ B cells demonstrated different gene expression profile from healthy controls. The 401 DE transcripts, in particular, the 71 genes uniquely expressed in RA and the 17 genes uniquely expressed in HC clearly distinguish active RA patients from healthy individuals. These DE genes were highly enriched in the chemokine/cytokine activity and chemokine/cytokine receptor binding GO systems and KEGG pathways, including CCL20, CCL21, CXCL1, CXCL2, CXCL10, and CCR7. These chemokines might attract more lymphocytes and granulocytes to the inflamed sites, thus perpetuating the proinflammatory status and the disease progression. Yet, these DE genes need to be further validated using larger candidate samples in the following studies.

In summary, here we demonstrated that CD27^+^IgD^+^ B cells were numerically and functionally impaired in RA with altered BCR repertoire and gene signature, which might contribute to the disease progression. Modulating the status of CD27^+^IgD^+^ B cells might provide novel therapeutic strategies for RA.

## Ethics Statement

This study was carried out in accordance with the recommendations of “Institutional Medical Ethics Review Board of Peking University People’s Hospital.” All subjects gave written informed consent in accordance with the Declaration of Helsinki. The protocol was approved by the “Institutional Medical Ethics Review Board of Peking University People’s Hospital.”

## Author Contributions

Conceived and designed the experiments: FH and ZL. Performed the experiments: FH and WZ. Analyzed the data: FH, WZ, YW, and ZL. Contributed reagents, materials and analysis tools: LS, XL, YJ, LX, HZ, YL and JQ. Wrote the manuscript: FH. Reviewed and edited the manuscript: DX, LL, XQ, and WL.

## Conflict of Interest Statement

The authors declare that the research was conducted in the absence of any commercial or financial relationships that could be construed as a potential conflict of interest.
