# Supplementary material for: Impaired CD27+IgD+ B Cells With Altered Gene Signature in Rheumatoid Arthritis
Source: Front Immunol. 2018 Mar 23;9:626. doi: 10.3389/fimmu.2018.00626 (PMC5877504; doi:10.3389/fimmu.2018.00626)
Supplement: Supplementary file 1 [file Table_1.docx]

**Supplementary Table 1 Primers used in the study.**

| **Genes** | **Sense primer (5**′**-3**′**)** | **Antisense primer (5**′**-3**′**)** |
| --- | --- | --- |
| GAPDH  IgM  IgM VH | AAGGTGAAGGTCGG AGTCAA  GCTGAGGCAAAGGAGTCTG  VH3:  GAGGTGCAGCTCGAGGAGTCTGGG  VH4: CAGGTGCAGCTCGAGCAGTCTGGG  VH5: CAGGTACAGCTCGAGCAGTCAGG  VH6: CAGGTGCAGCTGCTCGAGTCGGG | AATGAAGGGGTCATTGATGG  TGGTCTGCTTCAGTGGCG  CµCH1 (first round):  ACGCTGCTCGTATCCGACGGG  J2 (second round):  GTGACCAGGGTNCCTTGGCCCCAAG |
